# Natural History and Factors Associated with Early and Delayed Mortality in HIV-Infected Patients Treated of Tuberculosis under Directly Observed Treatment Short-Course Strategy: A Prospective Cohort Study in India

**DOI:** 10.1155/2012/502012

**Published:** 2012-12-18

**Authors:** Gerardo Alvarez-Uria, Praveen Kumar Naik, Raghavakalyan Pakam, Lakshminaryana Bachu, Manoranjan Midde

**Affiliations:** Department of Infectious Diseases, Bathalapalli Rural Development Trust Hospital, Kadiri Road, Anantapur District Bathalapalli, Andhra Pradesh 515661, India

## Abstract

Despite the impressive global results of DOTS in India, the effectiveness of DOTS for the treatment of tuberculosis in HIV-infected patients is not well known. This is an observational prospective cohort study performed in Anantapur District, Andhra Pradesh, India. The study included 1000 DOTS antituberculosis treatment (ATT) episodes and 840 person-years. CD4 lymphocyte count was below 200 cells/mm^3^ in 77% of the cases, and 21% were retreatments. Two thirds were presented with extrapulmonary tuberculosis, and the most common form of extrapulmonary tuberculosis was tuberculous meningitis followed by pleuritis, abdominal tuberculosis, and lymphadenitis. Cumulative incidence of mortality was 16%, 26%, 39%, and 46% at 1, 3, 12, and 24 months, respectively. Factors associated with three-month (early) mortality were being homeless, having low CD4+ lymphocyte count, having tuberculous meningitis, belonging to a socially disadvantaged community, having more than 35 years, and being on an antiretroviral therapy at the moment of initiating the ATT. Factors associated with delayed mortality were having low CD4+ lymphocyte count, belonging to a socially disadvantaged community, receiving a category II ATT because of a previous episode of ATT and having acid fast bacilli in sputum before the ATT initiation. These findings indicate that there is an urgent need to improve the treatment of tuberculosis in HIV-infected patients in India.

## 1. Introduction

Tuberculosis is a major public health problem worldwide. Although tuberculosis is a treatable disease, there were 8.8 million cases of tuberculosis and 1.45 million deaths from tuberculosis in 2010 [1]. With an incidence of 2.3 million cases, India has the highest burden of tuberculosis in the world, and one out of every four cases of tuberculosis worldwide occurred in India in 2010 [1]. India is also the third country in the world in terms of number of people infected by HIV, and 9% of patients with tuberculosis who are tested of HIV are HIV-infected [1, 2]. 

HIV and tuberculosis form a deadly synergy. Latent tuberculosis is common in developing countries, and the immunodeficiency produced by HIV increases the risk of developing active tuberculosis infection [3]. Moreover, HIV modifies the clinical presentation and the prognosis of tuberculosis. Patients with HIV infection have higher risk of extrapulmonary tuberculosis, tuberculosis relapse, and death than non-HIV-infected patients [4].

Treatment of tuberculosis in India has been implemented under the Revised National Control Tuberculosis Programme (RNTCP). RNTCP follows the standard direct observed treatment short-course (DOTS) strategy recommended by the World Health Organization (WHO) in 2003 [5]. Although the DOTS strategy has achieved impressive results in India [6], DOTS was endorsed by WHO based on observational studies performed in non-HIV-infected patients [5]. Despite the high burden of HIV-related tuberculosis and the high mortality of tuberculosis in HIVinfected patients, data about the effectiveness of DOTS programmes in HIV-infected people are scarce [7, 8]. The aim of this study was to evaluate the natural history of HIV-infected patients who were treated of tuberculosis under DOTS in a large HIV cohort from India.

## 2. Methods

This study was performed in the district of Anantapur, Andhra Pradesh. Andhra Pradesh is the state with highest burden of HIV in India [9]. Trends of the HIV epidemic in Anantapur have been described elsewhere [10]. Rural Development Trust (RDT) is a nongovernmental organization that has three hospitals in Anantapur. In these hospitals, medical care of HIV-infected people is provided free of cost, including medicines and consultation or admission charges. The Vicente Ferrer HIV Cohort Study (VFHCS) is an open cohort study of all HIV-infected patients visited in RDT hospitals [11]. Since September 2009, clinical information of the patients is collected prospectively. For this study, all antituberculosis treatment (ATT) episodes of patients from the VFHCS database from September 1st 2009, to September 1st 2011, were included in the analysis. The selection of patients from the database was executed in May 23rd 2012.

All HIV-infected patients with suspicion of tuberculosis infection were admitted to the hospital for performing acid fast bacilli (AFB) staining of sputum, chest radiograph, and, if clinically relevant, analysis of cerebrospinal fluid, pleural fluid, or ascitic fluid. Cryptococcus antigen was performed in all cerebrospinal fluid specimens to rule out cryptococcal meningitis. In smear-negative patients referring important weight loss but no other clinical sign of tuberculosis infection, an abdominal ultrasound was performed for investigating signs of abdominal tuberculosis [12, 13]. Following WHO recommendations for the definition of tuberculosis case [14], diagnosis of tuberculosis was based on the clinical judgment of the treating physician or a combination of the clinical judgment plus the presence of acid fast bacilli (AFB) and/or caseating or necrotizing granuloma in clinical specimens, in addition to a clinical response to ATT [15]. Disseminated tuberculosis was defined when there were signs of tuberculosis infection in two different sites. Initially, patients were initiated on ATT in the hospital, and, once they were stabilized, patients were referred to take ATT under RNTCP, which provides antituberculosis drugs free of cost through a decentralized network of primary healthcare facilities. The RNTCP follows the standard WHO DOTS strategy [5], which provides antituberculosis drugs three times a week during six months (category I) for patients who initiate ATT for the first time and during 8 months (category II) for patients who had previous ATT for at least one month or patients who experienced category I failures. Drugs were not given in fixed drug combinations as each drug was provided in a single formulation. For category I treatment, rifampicin, isoniazid, pyrazinamide, and ethambutol were given for two months, followed by rifampicin and isoniazid for four months [16]. For category II treatment, patients received streptomycin, rifampicin, isoniazid, pyrazinamide, and ethambutol for two months, rifampicin, isoniazid, pyrazinamide, and ethambutol for one month, and, finally, rifampicin, isoniazid and ethambutol for five months [16]. 

Information about patients' community was collected by self identification of the patients. Scheduled caste community is the lowest caste in the traditional Hindu caste hierarchy and, therefore, suffers social and economic exclusion and disadvantage. Scheduled tribe community is generally geographically isolated with limited economic and social contact with the rest of the population. Scheduled castes and scheduled tribes are considered a socially disadvantaged communities and are supported by positive discrimination schemes operated by the Government of India [17]. Backward castes form a collection of “intermediate” castes that were considered low in the traditional caste hierarchy, but above scheduled castes.

Statistical analysis was performed using Stata Statistical Software (Stata Corporation. Release 11. College Station, Tx, USA). We used Kaplan-Meier survival curves and the log-rank test for comparing factors associated with mortality. Time was measured from the date of the ATT initiation to death or the last visit date. Multivariable analysis was performed with Cox regression proportional hazard models. Because CD4+ lymphocyte count was not available for 32 cases, missing values were imputed using the stratified hot deck imputation implemented for Stata by Martin Schonlau (hotdeckvar command) [18]. The proportional hazard assumption was assessed performing log-log survival curves and statistical tests based on Schoenfeld residuals [19]. The goodness of fit of the models was assessed using the Harrell's C concordance statistic [20]. Because the proportional hazard assumption was violated with the initial global model, which included survival estimates from the initiation of ATT to the death or last visit date, we decided to build two different Cox regression models, one for studying factors associated with death during the first three months after initiating ATT (early mortality) and another one for studying factors associated with death after completing three months of ATT (delayed mortality) [19]. Two-way interactions for both early and delayed mortality models were checked. In both models, we found that gender had interactions with other variables, so finally we performed global and stratified by gender models of early and delayed mortality [21]. The study was approved by the ethical committee of the RDT Institutional Review Board.

## 3. Results

During the study period, we identified 1000 ATT episodes. Characteristics and median CD4+ lymphocyte count of patients at the moment of initiating ATT are presented in Table 1. The median age was 35 years (interquartile range (IQR), 30–41), and the median CD4+ lymphocyte count was 116 cells/mm^3^ (IQR, 61–190). Almost two thirds of the patients were male, more than a half were illiterate, 4.6% were homeless, and 31.2% belonged to a socially disadvantaged communities. One fourth of patients were smear positive, one fifth received category II ATT, and 38% had started ART before the initiation of ATT. Of 590 patients who were alive after six months of ATT, 91 (15.4%) had not initiated ART. The most common form of tuberculosis infection was pulmonary tuberculosis followed by meningitis, pleuritis, abdominal tuberculosis, lymphadenitis, and disseminated tuberculosis. 

**Figure 1:**
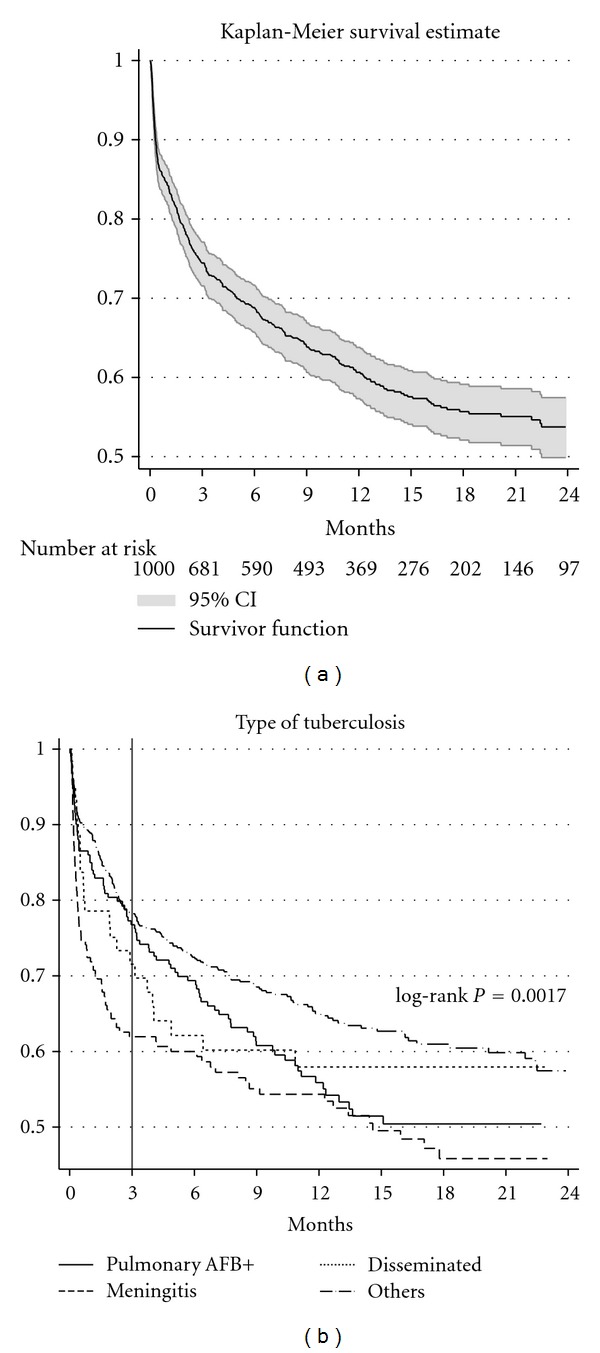
Kaplan-Meier survival curves of all patients and by type of tuberculosis. AFB: acid fast bacilli; CI: confidence interval.

**Figure 2:**
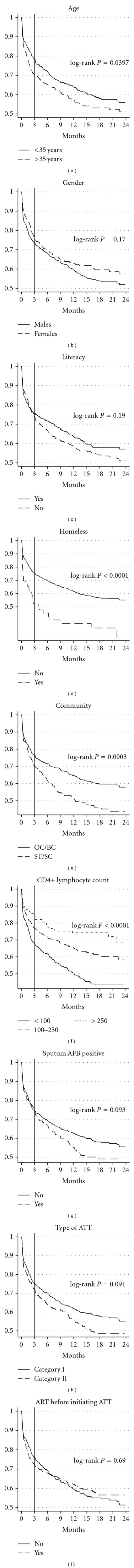
Kaplan-Meier survival curves by age, gender, literacy, being homeless, community, CD4+ lymphocyte count (cells/mm^3^), presence of AFB+ sputum, type of antituberculosis treatment, and timing of the antiretroviral therapy. (AFB: acid fast bacilli; ART: antiretroviral therapy; ATT: antituberculosis treatment; OC/BC: nondisadvantaged communities: other castes and backward castes; ST/SC, disadvantaged communities: scheduled tribes, scheduled castes.)

The study involved 840 person-years with a mean followup of 10.1 months and 388 deaths. The cumulative incidence of mortality was 15.7% (95% confidence interval (CI), 13.5–18.1) at 1 month, 25.6% (95% CI, 22.9–28.5) at 3 months, 31.2% (95% CI, 28.3–34.3) at 6 months, 39.4% (95% CI, 36.2–42.7) at 12 months, and 46.3% (95% CI, 42.5–50.1) at 24 months. The Kaplan-Meier survival curve with number at risk is presented in Figure 1. Also in Figure 1, we can observe the survival curves of the different types of tuberculosis infections. Patients with meningitis and disseminated tuberculosis had higher incidence of mortality during the first three months of ATT. The incidence of mortality was reduced after three months of ATT except in patients with smear-positive pulmonary tuberculosis, where the cumulative incidence of mortality remained more or less constant until approximately 14 months after the initiation of ATT. Kaplan-Meier survival curves with log-rank test for other covariates are presented in Figure 2. Intuitively, differences among the covariates age, being homeless, and CD4+ lymphocyte count were visible since the initiation of ATT, whereas differences among the covariates gender, literacy, community, and having AFB positive sputum were more visible after three months of ATT. Although there was not an important difference in the final survival, patients who were on ART at the moment of initiating ATT had slightly higher mortality during the first three months of ATT and slightly lower mortality thereafter.

Multivariable analysis of factors associated with death during the first three months of ATT is presented in Table 2. In descending order by hazard ratio, factors associated with increased risk of early mortality were being homeless, having low CD4+ lymphocyte count, having tuberculous meningitis, belonging to a socially disadvantaged caste, having more than 35 years, and being on ART at the moment of initiating ATT. The main difference between men and women was that, in women, belonging to a disadvantaged caste was not a so strong factor for early mortality as in men.

Multivariable analysis of factors associated with death after three months of ATT is presented in Table 3. In descending order by hazard ratio, factors associated with increased risk of delayed mortality were having low CD4+ lymphocyte count, belonging to a socially disadvantaged caste, receiving category II ATT because of a previous episode of ATT, and having AFB positive sputum. The main difference between men and women was that, in women, belonging to a disadvantaged caste was a less important factor for delayed mortality than being illiterate. Although not statistically significant, being on ART at the initiation of ATT was a protective factor for delayed mortality.

## 4. Discussion 

This study describes the high mortality of HIV-infected patients who received ATT under DOTS strategy in this district of India. In Pune, India, a 51% mortality after 30 months of followup in 121 HIV-infected patients who received ATT under RNTCP was observed [7]. These mortality rates are higher than the ones described in the observational studies from Sub-Saharan Africa and other developing countries [22–25] indicating that there is an urgent need to improve the treatment of tuberculosis for HIV-infected patients in India. Although we did not collect information about adherence to ATT, previous studies in India have shown that a high number of patients under “directly observed” RNTCP regimens are poor or nonadherent to treatment [26, 27]. Besides interventions to support the completion and the adherence of ATT, there is a need to improve current guidelines for treating tuberculosis in HIV-infected patients according to recent evidence [28]. Current WHO guidelines strongly recommend daily ATT regimen for HIV-infected patients, especially during the intensive phase [14], because daily ATT regimens have higher cure rates and lower risk of relapse and treatment failure compared to intermittent ATT regimens [29]. In a randomized trial performed with HIV-infected patients from India with a followup duration of 36 months, patients treated with intermittent category I treatment had 36% mortality, 15% bacteriological recurrence, and all patients who experienced treatment failure developed acquired rifampicin resistance [8]. This mortality is lower than the mortality found in our study, but moribund patients were excluded from the study, whereas we included all patients admitted to the hospital. In this randomized trial, extending intermittent treatment for three extra months did not improve mortality nor acquired rifampicin resistance but reduced by half the number of bacteriological recurrences. Also recommended by WHO, the use of fixed dose combinations could improve the adherence to ATT [14]. New interventions for achieving early diagnosis of tuberculosis and HIV can have a dramatic impact in reducing the mortality of HIV-related tuberculosis. In India, still 40–50% of HIV-infected patients are diagnosed of HIV when their CD4+ lymphocyte count is below 200 cells/mm^3^, and, in many cases, tuberculosis is the first manifestation of HIV infection [30, 31]. In our study, the CD4+ lymphocyte count was less than 200 cells/mm^3^ in 77% of the cases. If patients could be diagnosed of HIV with higher CD4+ lymphocyte count, ART could be started before the appearance of tuberculosis, averting the transmission of tuberculosis to others as well [3].

In accordance with previous studies [32], the socioeconomic characteristics of the patients were important factors related to early and delayed mortality. In our study, homeless patients and people from disadvantaged communities had higher risk of death, especially men. As seen before in India [33], receiving category II ATT because of previous treatment of tuberculosis was associated with delayed mortality. Interestingly, the type of tuberculosis infection was an important factor for early mortality but not for delayed mortality, whereas having AFB positive sputum was an important factor for delayed mortality but not for early mortality. Among all types of tuberculosis, tuberculous meningitis had the highest incidence of death during the first three months of ATT and was an independent factor related to early mortality after adjusting by other variables.

Being on ART at the moment of initiating ATT was associated with increased early mortality. It is probable that the immunity recovery after the initiation of ART provokes an increased inflammatory response against the high mycobacterial organism load present in HIV-infected patients, unmasking the tuberculosis infection and increasing the risk of death due to the strong inflammatory reaction [4]. In Haiti, patients diagnosed of tuberculosis during the first three months of ART were 3.25 times more likely to die than other HIV-infected patients with tuberculosis [34]. According to these findings, patients who are going to initiate ART should undergo a complete evaluation to rule out tuberculosis before the initiation of ART.

The study has some limitations. Diagnosis of tuberculosis was performed according to the definition of tuberculosis case suggested by WHO [14], but it was not confirmed by culture of mycobacteria. It is possible that some patients were wrongly diagnosed of tuberculosis infection. However, in most resource-limited settings, mycobacterial culture is not available, so including only those patients with a positive culture for *Mycobacterium tuberculosis* would not reflect the common clinical practice in the management of tuberculosis in developing countries. We did not perform drug sensitivity testing so it is possible that the presence of resistant tuberculosis could explain some of the deaths observed in the study. However, the proportion of patients having rifampicin resistance is only 2.2% in our area [35], so the possibility of a bias because of high prevalence of multidrug-resistant tuberculosis in unlikely. In addition, we did not have information about the adherence of patients to ATT or the proportion of patients who defaulted from treatment or were lost to followup, which are very important factors for the success of DOTS [32]. 

## 5. Conclusions 

The results of this study show the high mortality of HIV-infected patients treated of tuberculosis under DOTS in this area of India, confirming what was observed in previous studies from other parts of India. These patients are diagnosed of tuberculosis with low a CD4+ lymphocyte count and a high proportion of them present with extrapulmonary tuberculosis. When investigating factors associated with mortality, it is useful to differentiate between factors associated with early mortality and factors associated with delayed mortality. We found that tuberculous meningitis and being on ART when initiating ATT are associated with early mortality. Factors related to poor socioeconomical status such as being homeless or belonging to a disadvantaged community are also associated with an increased risk of death. Patients who received ATT previously and patients having AFB positive sputum have higher risk of delayed mortality. These findings indicate that there is an urgent need to improve the treatment of tuberculosis in HIV-infected patients in India.

## Figures and Tables

**Table 1:** General characteristics of the patients and the median CD4+ lymphocyte count.

	*N*	% (95% CI)	Median CD4 count* (IQR)
Gender			
Male	659	65.9 (62.9–68.8)	113 (58–183)
Female	341	34.1 (31.2–37.1)	124 (67–212)
Literacy			
Yes	410	41 (38–44.1)	115 (56–188)
No	590	59 (55.9–62)	116 (65–195)
Homeless			
No	954	95.4 (93.9–96.5)	115 (60–189)
Yes	46	4.6 (3.5–6.1)	133 (74–214)
Community			
Scheduled tribe	77	7.7 (6.2–9.5)	115 (60–189)
Scheduled cast	235	23.5 (21–26.2)	124 (64–207)
Backward cast	490	49 (45.9–52.1)	123 (66–189)
Other casts	198	19.8 (17.4–22.4)	104 (53–181)
AFB in sputum			
Negative	743	74.3 (71.5–76.9)	118 (66–194)
Positive	257	25.7 (23.1–28.5)	109 (50–181)
Previous ATT			
No (category I)	794	79.4 (76.8–81.8)	115 (61–183)
Yes (category II)	206	20.6 (18.2–23.2)	126 (61–219)
Organ involved			
Abdominal	117	11.7 (9.8–13.8)	117 (64–175)
Lymphadenitis	89	8.9 (7.3–10.8)	137 (74–217)
Meningitis	190	19 (16.7–21.6)	115 (64–181)
Pleuritis	174	17.4 (15.2–19.9)	132 (67–204)
Pulmonary AFB negative	137	13.7 (11.7–16)	105 (52–195)
Pulmonary AFB positive	205	20.5 (18.1–23.1)	115 (60–200)
Disseminated	63	6.3 (4.9–8)	103 (39–178)
Others	25	2.5 (1.7–3.7)	135 (84–207)
Timing of ART			
Before ATT	384	38.4 (35.4–41.5)	153 (86–218)
0-1 months after ATT	176	17.6 (15.4–20.1)	103 (49–147)
1-2 months after ATT	91	9.1 (7.5–11.1)	109 (60–197)
2–6 months after ATT	57	5.7 (4.4–7.3)	109 (42–183)
No ART	292	29.2 (26.5–32.1)	95 (47–165)

AFB: acid fast bacilli; ART: antiretroviral therapy; ATT: anti-tuberculosis treatment; CI: confidence interval; IQR: interquartile range. *cells/mm^3^.

**Table 2:** Cox regression analysis of factors associated with death during the first three months of anti-tuberculosis treatment.

	Overall		Males		Females	
	HR (95% CI)	*P* value	HR (95% CI)	*P* value	HR (95% CI)	*P* value
Age (years)						
<35	1		1		1	
>35	1.37 (1.06–1.77)	0.017	1.24 (0.91–1.68)	0.179	1.83 (1.14–2.94)	0.013
Literacy						
Yes	1		1		1	
No	1.08 (0.82–1.41)	0.593	0.98 (0.72–1.34)	0.917	1.5 (0.75–2.98)	0.248
Homeless						
No	1		1		1	
Yes	2.6 (1.65–4.08)	<0.001	2.57 (1.34–4.93)	0.004	3.29 (1.69–6.42)	<0.001
Community						
ST/SC	1.41 (1.08–1.84)	0.011	1.58 (1.15–2.16)	0.005	1.06 (0.64–1.75)	0.822
OC/BC	1		1		1	
CD4+ lymphocyte count*						
<100	2.22 (1.4–3.52)	0.001	1.74 (0.99–3.04)	0.053	3.09 (1.35–7.09)	0.008
100–250	1.52 (0.95–2.42)	0.08	1.2 (0.69–2.11)	0.521	2.14 (0.92–4.95)	0.077
>250	1		1		1	
Previous ATT						
No (category I)	1		1		1	
Yes (category II)	1.2 (0.88–1.63)	0.251	1.15 (0.8–1.65)	0.452	1.32 (0.73–2.38)	0.364
Organ involved						
Meningitis	1.82 (1.37–2.43)	<0.001	2.02 (1.45–2.83)	<0.001	1.21 (0.67–2.21)	0.526
Disseminated	1.43 (0.87–2.38)	0.162	1.45 (0.82–2.55)	0.197	1.5 (0.46–4.84)	0.5
Others	1		1		1	
ART before ATT						
No	1		1		1	
Yes	1.32 (1.01–1.73)	0.043	1.28 (0.92–1.77)	0.138	1.46 (0.89–2.39)	0.135
Gender						
Male	1.12 (0.83–1.5)	0.456				
Female	1					

AFB: acid fast bacilli; ART: antiretroviral therapy; ATT: anti-tuberculosis treatment; CI: confidence interval; HR: hazard ratio. *cells/mm^3^.

**Table 3:** Cox regression analysis of factors associated with death after three months of anti-tuberculosis treatment.

	Overall		Males		Females	
	HR (95% CI)	*P* value	HR (95% CI)	*P* value	HR (95% CI)	*P* value
Age (years)						
<35	1		1		1	
>35	0.92 (0.66–1.29)	0.633	1.14 (0.76–1.71)	0.523	0.61 (0.31–1.18)	0.143
Literacy						
Yes	1		1		1	
No	1.28 (0.9–1.84)	0.172	1.04 (0.69–1.56)	0.868	2.78 (0.98–7.91)	0.055
Homeless						
No	1		1		1	
Yes	1.8 (0.86–3.76)	0.121	1.96 (0.69–5.53)	0.206	1.67 (0.56–4.94)	0.358
Community						
ST/SC	1.87 (1.32–2.64)	<0.001	2.39 (1.54–3.69)	<0.001	1.49 (0.8–2.78)	0.208
OC/BC	1		1		1	
CD4+ lymphocyte count*						
<100	2.85 (1.61–5.04)	<0.001	2.53 (1.22–5.23)	0.012	3.23 (1.27–8.19)	0.014
100–250	1.58 (0.89–2.82)	0.121	1.43 (0.68–2.98)	0.344	1.72 (0.67–4.39)	0.258
>250	1		1		1	
Previous ATT						
No (category I)	1		1		1	
Yes (category II)	1.61 (1.09–2.39)	0.017	1.56 (0.99–2.45)	0.055	1.69 (0.78–3.67)	0.184
Sputum AFB						
Negative	1		1		1	
Positive	1.54 (1.08–2.18)	0.016	1.92 (1.26–2.91)	0.002	1.08 (0.53–2.21)	0.836
ART before ATT						
No	1		1		1	
Yes	0.76 (0.52–1.1)	0.147	0.91 (0.58–1.43)	0.688	0.54 (0.26–1.11)	0.094
Gender						
Male	1.1 (0.76–1.6)	0.618				
Female	1					

AFB: acid fast bacilli; ART: antiretroviral therapy; ATT: anti-tuberculosis treatment; CI: confidence interval; HR: hazard ratio. *cells/mm^3^.
